# Quality control procedure for Coccidial vaccines versus different routes of immunization

**DOI:** 10.14202/vetworld.2022.2342-2347

**Published:** 2022-09-29

**Authors:** Arwa Elnaggar, Hala Mahmoud, Sahar Saber

**Affiliations:** 1 Department of Parasitology, Central Laboratory for Evaluation of Veterinary Biologics, Agriculture Research Center (ARC), Cairo, Egypt; 2 Department of Inactivated Viral Poultry vaccines, Central Laboratory for Evaluation of Veterinary Biologics, Agriculture Research Center, Cairo, Egypt; 3 Department of Live Attenuated Viral Poultry Vaccines, Central Laboratory for Evaluation of Veterinary Biologics, Agriculture Research Center, Cairo, Egypt

**Keywords:** coccidia, *Eimeria tenella*, anticoccidial drugs and vaccinations, spray

## Abstract

**Background and Aim:**

Coccidiosis is an enteric infection caused by a protozoon (*Eimeria* *tenella*). Coccidiosis is known to have a negative impact on the economy. Coccidiosis is controlled using anticoccidial drugs, antibiotics, and vaccines. Various coccidial vaccines differ in application technique, attenuation method, and the species used. Coccidial vaccines can be spray or gel-based (Form). This study aimed to compare the effect of application and approaches between spray and gel vaccines for coccidiosis.

**Materials and Methods:**

Specific pathogen-free chicks were vaccinated with different vaccines. Fecal samples were taken on 21 days post-vaccination for vaccine take, and then a challenge test was done on day 21.

**Results:**

Post-vaccination oocyst counts in gel vaccinated groups were more than the spray vaccinated ones as it recorded (1400 and 2200) oocyst/g, but the gel vaccines resulted in lower post vaccinal titer which was (10000 and 12500) oocyst/g. Results of quantitative real-time polymerase chain reaction test post-vaccination were (23.72, 20.29) cycle threshold (CT) for spray vaccines and (18.75, 17.62) CT for gel vaccinated group. By challenging all the experimental groups, the microscopic and macroscopic lesion of gel vaccines resulted in score 1, while spray vaccines groups recorded score 2 and the control non-vaccinated challenged chickens showed score 4. The non-vaccinated/non-challenged group recorded a score of zero.

**Conclusion:**

These results can help poultry producers to decide which delivery system will provide the best results for their production system. The gel vaccines showed a better protection rate and lower shedding, which means more protection of birds and public health.

## Introduction

Avian coccidiosis is an infectious disease of small intestine caused by intracellular parasitic *Eimeria tenella* (phylum Apicomplexa). The coccidial infection is characterized by localized lesions leading to malnutrition, anorexia, hampered livestock performance, and high mortality rate [1]. It impairs digestive tract capacity causing poor absorption efficiency growth [2]. Avian coccidiosis is a major parasitic disease with a negative economic consequence on poultry production globally, which is supposed to lose about 3.2 billion dollars yearly [3]. The up-to-date control parameters measures involve live vaccines and anticoccidial drugs [4]. *Eimeria* life cycle includes two extracellular and intracellular stages eliciting a powerful inflammatory response accompanying excessive tissue damage due to lipid peroxidation, severe hemorrhagic diarrhea, secondary infection by other pathogenic agents and may lead to death [4].

Different anticoccidial drugs can be used to control coccidiosis, however, many disadvantages are reported such as drug residues in tissues. The scientific community is engaged in developing more safe and effective anticoccidial compounds [5]. There are seven global widespread known *Eimeria* species [6, 7], namely, *E. tenella, Eimeria brunetti, Eimeria maxima, Eimeria praecox, Eimeria necatrix, Eimeria mitis*, and *Eimeria acervulina*. *Eimeria* infection leads to poor growth performance due to imperfect intestinal activity [8, 9]. The disease is characterized by resistance to anticoccidial drugs [10] and asymptomatic manifestation [6, 11]. Coccidiosis may also be associated with intestinal colonization of other bacteria such as Clostridium and *Salmonella*, leading to additional economic losses [12, 13]. The mortality rate could be 34.8% [14]. The primary diagnostic factor is finding discharged oocysts in feces.

Coccidiosis is being controlled using live vaccines [15]. The basic component in all vaccines is sporulated oocysts from various species, primarily *E. tenella*, *E. maxima*, and *E. acervulina*. There are various vaccine administration techniques, such as spraying and applying gel droplets to diet [16–18]. Coccidia spray vaccines are commonly administered to one-day-old chicks [19].

The previous studies evaluated the impact of using different coccidia vaccine application techniques by comparing the number of excreted coccidia oocyst and the protective efficacy of each route [16, 20, 21]. The studies have reached various conclusions regarding the level of protection of different application methods and concluded that the difference mainly lies in the number of oocysts shed between different delivery methods. Hence, the present study was performed to evaluate the most protective and effective application technique. The current research will be helpful in improving Eimeria vaccine programs using the right dose in the right application method. Thus, improving the flock’s health and growth rate, following vaccination.

This study aimed to compare the effect of application and approaches between spray and gel vaccines for coccidiosis.

## Materials and Methods

### Ethical approval

The study was approved ethically by Central Laboratory for Evaluation of Veterinary Biologics (CLEVB), Cairo, Egypt.

### Study period and location

The study was conducted from August 2020 to July 2021. The laboratory works were conducted at Central Laboratory for Evaluation of Veterinary Biologics (CLEVB), Agriculture Research Center, Cairo, Egypt.

### Vaccines

Four batches of live attenuated anticoccidial vaccines were used to vaccinate four groups of 1-day old chicks, two of them were administered as a gel (Coccivac-B52 spray contains *E. acervulina*, *E. maxima*, *E. mivati*, and *E. tenella*) and the other two were applied as spray delivery system (Immucox III gel contains *E. acervulina*, *E. maxima*, and *E. tenella*) [19].

### Oocyst counting

Oocysts were counted by microscopic enumeration technique using McMaster method. Briefly, each vaccine was mixed to its suitable diluents according to manufacturer’s instructions. The Oocysts were counted to be typed by the morphological characters of each species included in each vaccine [22].

### Vaccination and experimental design

One-day-old specific pathogen-free chicks were divided into five groups (40 of each) and kept in separate isolators. On the 5^th^ day, groups (2) and (3) were vaccinated by gel vaccine (Immucox III gel contains *E. acervulina*, *E. maxima*, and *E. tenella*), groups (4), and (5) were vaccinated by spray vaccine (Coccivac-B52 spray contains *E. acervulina*, *E. maxima*, *E. mivati*, and *E. tenella*) while Group (1) was non-vaccinated control group. Fecal samples were taken at 21 days post-vaccination (DPV) and each group was subdivided into four subgroups for *Eimeria* species-specific challenge.

### Vaccine reaction

On the 21^st^-DPV, the vitality of the vaccine was established, and fecal samples were collected to measure the first oocyst shedding.

On the 21^st^-DPV, 10 vaccinated chicks of each group were euthanized for careful inspection of serosal and mucosal surfaces of the gut gross lesions and scarpings at the affected sites. The gross lesions were more observed in the midgut by the appearance of Meckel’s diverticulum. Scores were recorded from 0 to 4, 0 means no lesions present, 1 means little serosal petechiae of intestine, 2 means more petechiae, 3 means intestinal wall thickening, and score 4 means bloody contents in intestine.

The collected samples also were used for microscopical detection of the parasites’ developing stages [23]. The vaccine is considered satisfactory if 90% of vaccinated chicks had positive oocyst with local mucosal lesions.

### Quantitative real-time polymerase chain reaction (RT-PCR)

Twenty-one days post-vaccination and post-challenge, fecal samples were collected for detection and quantification of *Eimeria* spp. as shown in Table-1.

**Table-1:** Universal coccidia primers sequences for real-time PCR for detection of *Eimeria* and cycling conditions.

Target gene	Primers sequences	Amplified segment (bp)	Primary denaturation	Amplification (40 cycles)			Dissociation curve (1 cycle)		
	
				Secondary denaturation	Annealing	Extension	Secondary denaturation	Annealing	Final denaturation
*Eimeria* ITS1	GCAAAAGTCGTAACACGGTTTCC	500	94°C 5 min	94°C 30 s	56°C 40 s	72°C 45 s	94°C 1 min	56°C 1 min	94°C 1 min
	CTGCAATTCACAATGCGTATCG

PCR=Polymerase chain reaction

### DNA extraction

Total DNA from the 220 mg fecal samples was extracted by following the manufacturer’s instructions (QIAamp DNA stool Mini Kit, QIAGEN, Hilden, Germany).

### Real-time polymerase chain reaction

The PCR amplification was carried out using coccidial primers (Table-1) in a 25 μL reaction with 12.5 μL of 2× QuantiTect Master Mix (QIAGEN), 0.5 μL (20 pmol) of each primer, 8.5 μL of water, and 3 μL of DNA template. The reaction was performed in an MX3005P real-time PCR machine [24] (Agilent, CA, USA).

## Results

### Vaccine reaction

The first oocyst shedding counts in the collected fecal samples on 21 DPV are shown in Table-2. Results showed that more than 90% of vaccinations were successfully administered for each test group. The oocyst count of gel vaccines was higher than the spray vaccine. Group (4) recorded 1400 oocyst/g and group (5) recorded 2200 oocyst/g, while the groups vaccinated with gel vaccines (2,3) recorded higher titer of 10000 and 12500 oocyst/g, respectively. These results were confirmed by the gross and microscopical lesion scores which were <2 for all vaccinated groups. All control chickens were free from any gross intestinal lesions.

**Table 2:** Oocyst counting per mL using traditional method 21st day post-vaccination measured by oocyst/g.

Chicken group	Sample count
Group 1 (control)	-
Group 2 (gel)	10000
Group 3 (gel)	12500
Group 4 (spray)	1400
Group 5 (spray)	2200

### Quantitative RT-PCR

**Figure-1:**
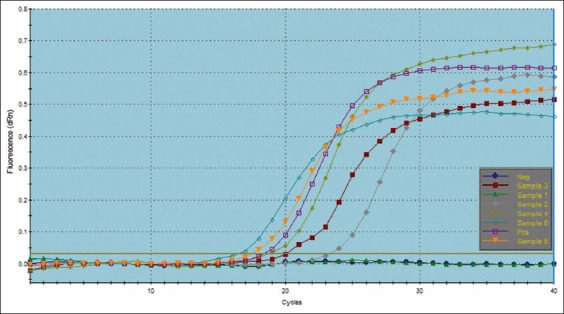
The amplification plots of real-time polymerase chain reaction for *Eimeria* strain live vaccine post-vaccination.

**Figure-2:**
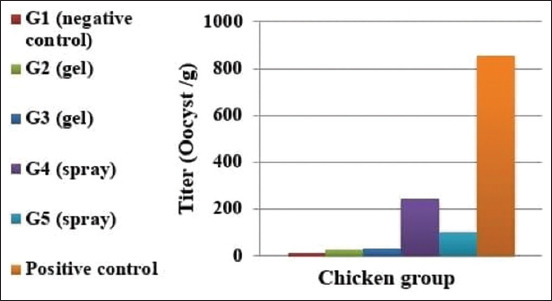
Mean oocyst titer of the real-time polymerase chain reaction for *Eimeria* strain post-vaccination.

According to the quantitative RT-PCR analysis, the groups who received the gel vaccine (Groups 2 and 3; cycle threshold [CT] values of 18.75 and 17.62, respectively) showed greater Eimeria shedding than the groups that received the spray vaccine (Groups 4 and 5; CT values of 23.41 and 20.29 CT, respectively) (Table-3, Figures-1 and 2). Scores for the gel and spray vaccination groups were 1 and 2, respectively. Both scores were lower than the control non-vaccinated challenged group’s score of 4, which was the highest.

**Table-3:** Mean CT values of the real-time PCRs for *Eimeria* strain post-vaccination.

Chicken group	Result	CT
Group 1 (control)	-	No CT
Group 2 (gel)	+	18.75
Group 3 (gel)	+	17.62
Group 4 (spray)	+	23.41
Group 5 (spray)	+	20.29
Positive control	+	16.95

*CT=Cycle threshold, *PCR=Polymerase chain reaction

### Oocyst count scores post challenge

The control challenged group recorded score 4, while the Eimeria Oocyst enumeration following intestinal scraping post challenge showed the same scores of gross lesion as the gel vaccine group recorded score 1 and the spray group gave score 2. The group that was not vaccinated or challenged received a score of 0.

### Quantitative RT-PCR post challenge

**Figure-3:**
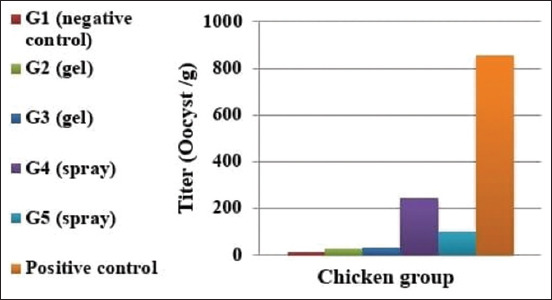
Mean oocyst titer of the real-time polymerase chain reaction for *Eimeria* strain post-challenge.

Using RT-PCR, the shedding of the *Eimeria* post challenge for groups from 1 to 5 was estimated. A very weak *Eimeria* shedding was recorded for Groups 2 and 3 (gel vaccine delivered groups) with CT values of 25.34 and 23.90, respectively. On the other hand, spray-vaccinated Groups 4 and 5 showed *Eimeria* shedding CT values of 48.38 and 41.72, respectively (Table-4 and Figure-3).

**Table-4:** Mean CT values of the real-time PCRs for *Eimeria* strain post-challenge.

Chicken group	Result	CT
Group 1 (control)	-	No CT
Group 2 (gel)	+	25.34
Group 3 (gel)	+	23.90
Group 4 (spray)	+	48.38
Group 5 (spray)	+	41.72
Positive control	+	16.95

CT=Cycle time, PCR=Polymerase chain reaction

## Discussion

Due to chemotherapeutic expenses and the upcoming threats attributed to antibiotics, different approaches to coccidiosis control are being studied. Coccidial vaccines are a well-known control procedure to control coccidiosis and overcome possible losses in poultry farms as the vaccine offers considerable immunity to future exposure of the same *Eimeria* spp. [25, 26]. In the present study, the influence of different application methods was estimated by the total count of Emeria oocyst shedding post-vaccination and post-challenge. The gel vaccinated groups showed higher numbers of fecal oocysts count on 21 DPV compared with spray vaccinated groups. The higher fecal oocyte count in gel vaccinated groups could be attributed to the ability of birds to ingest higher doses of oocysts following the vaccine application. On the other hand, the spray-vaccinated groups could not receive all sporulated oocysts, which in turn lead to low shedding in spray-vaccinated birds [21]. The quantitative PCR (qPCR) results are in agreement with traditional microscopic enumeration results [27].

In the present study, the qPCR is used as an alternative confirmatory method to count *Eimeria* oocyst as previously approved by Vrba *et al*. [28]. Using qPCR, a wide range of *Eimeria* oocysts of veterinary and public health importance can be detected and the technique is suitable for both routine research and diagnostic purposes. It was used in other studies as an approved and accurate screening technique for samples targeting *Eimeria* spp. [29]. Both the quantification methods, either traditional or qPCR, are reproducible and sensitive [30].

Vaccination of chicks by gel application shed approximately seven-fold more oocysts than chicks vaccinated by spray and the results are in agreement with a previous study which reported 12-fold more oocyst shedding for gel vaccine post-vaccination [31].

The oocyst shedding post-vaccination was explained mainly due to the transient mild coccidial infections induced by the vaccine and lesions of intestinal epithelium [16, 32]. Neither gross nor microscopic results post-vaccination differ in vaccinated chickens. The mild effect was attributed to a balanced supplied diet which supports the immune system [33]. The high oocyst count post vaccination demonstrated gel vaccines provide a more appropriate application method compared to the spray delivery technique [16].

Vaccines ingestion and effectiveness are affected by many factors such as vaccine administration method, the surrounding temperature, light, and sound intensity [34]. These factors affect the vaccines’ preening behavior by prohibiting some chickens from receiving the proper vaccine dose resulting in unexposed chickens to *Eimeria* post-vaccination [35].

A lowered humidity leads to decreased sporulation rates of the oocysts shed with consequently lower doses of oocysts ingestion by birds [21]. Therefore, the challenge test to evaluate the protection of each vaccine is important. After the challenge, the most important measure of efficacy to consider are the decreased parasitic count, lowering oocyst titer, parasite transmission, and diminished clinical signs of coccidiosis. Based on the measuring oocyte titer, chicks vaccinated by gel application can ingest 3.3-fold more vaccine than the spray vaccinated chicks [31]. In the other study, it reached 6.5-fold higher for the gel vaccines than the spray vaccines [36]. These differences are attributed to a difference in the equipment characteristics and application method which affect oocyst concentration.

The recovery rate allows more comparison between the gel and spray vaccines. The recovery rate in chicks vaccinated by gel vaccines application was significantly better than in chicks vaccinated by spray vaccines. This finding is ascribed to the gel-droplet vaccines which were ingested in a considerable amount than spray vaccines. The gel vaccines seem to be ingested more uniformly among birds [31]. Similar findings were reported also reported in the previous studies [21].

Low shedding in gel vaccinated groups than spray groups after the challenge could be attributed to the loss of about 55% of the total oocyst ingested by spray vaccinated chicks. The different protocols for vaccine application affect gastrointestinal tract infections and improve *Eimeria* recovery. The recovery rate can reach up to 45% after vaccination by spray vaccine when compared to the gel types [31]. This rate agrees with the present study which recorded recovery of gel vaccinated groups four times better than the spray vaccines. The results are in agreement with the previous work which highlighted the effect of application method, which in turn directly affects the development of protective immunity as it is mainly influenced by the vaccine administration uniformity [37, 38].

## Conclusion

If vaccinated chicks receive the recommended dose, our approach can aid chicken breeders in controlling *Eimeria* species cycle. Consequently, improve the flock’s health and growth by improving the ingested vaccine volume and uniformity in application strategy. The improved vaccination will develop a powerful and quick immunological response. The gel vaccines achieved the best protection with low shedding to the surrounding environment. On the other hand, the non-vaccinated chicks were more likely to be infected when exposed to a natural infection as they lacked immunological protection.

## Authors’ Contributions

AE: Designed the study. HM: Performed the study. AE, HM, and SS: Drafted the manuscript. All authors have read and approved the final manuscript.

## Data Availability Statement

All data generated or analyzed during this study are included in this published article.
